# Draft Genome Sequence of *Pseudomonas* sp. Strain CMAA 1215, a Plant Growth-Promoting Bacterium Isolated from a Brazilian Mangrove

**DOI:** 10.1128/genomeA.00995-13

**Published:** 2013-11-27

**Authors:** Rafael L. F. Vasconcellos, Rodrigo Mendes, Rodrigo G. Taketani, Tiago D. Zucchi, Itamar Soares Melo

**Affiliations:** Laboratory of Environmental Microbiology, Embrapa Environment, Jaguariúna, São Paulo, Brazil

## Abstract

The aim of this study was to sequence the genome of the plant growth-promoting *Pseudomonas* sp. strain CMAA 1215, an osmotolerant bacterium isolated from mangrove soil.

## GENOME ANNOUNCEMENT

Mangroves are remarkable environments found in tropical regions worldwide and have been considered a biodiversity hot spot (1). Brazil has one of the largest areas of mangroves in the world, and although it has been extensively studied for the ecological traits of its fauna and flora, there is still a lack of knowledge on its microbial communities. In our survey on the bacterial ecology of Brazilian mangroves, we recovered an osmotolerant bacterium, strain CMAA 1215, from the soil of the Cananéia Island mangrove (25°05′12.61″S, 47°57′41.21″W). This strain, classified as a *Pseudomonas* sp., has proven to be a potential plant growth promoter through its ability to produce 1-aminocyclopropane-1-carboxylate (ACC) deaminase and indolacetic acid (IAA) and solubilize inorganic phosphate.

Many members of the genus *Pseudomonas* have been described as plant growth promoters. However, new evidence has shown that *Pseudomonas* strains exhibit highly heterogenic genomes in which only 25 to 35% of the genes are shared by all members of the genus and about a third of the genes are unique to each strain (2). Hence, the discovery of new plant growth promoter genes may be advocated and exploited for the development of improvements regarding agricultural crop management.

Thus, to extend knowledge of the genes related to plant growth promotion in strain CMAA 1215, whole-genome sequencing was performed using the Ion Torrent (PGM) platform. Genomic DNA was extracted from a pure culture grown overnight on LB medium using the PureLink genomic DNA kit (Life Technologies). Sequencing was carried out on the Ion 316 chip sequencer provided in the Ion sequencing kit 200 bp version 2.0, according to the manufacturer’s protocol. The genome sequence was *de novo* assembled using the MIRA version 3.4, CLC Genomics Workbench version 5.5.1, and SeqMan NGen version 4.0.0 packages, and the contigs obtained from the assembly were integrated using CISA (3, 4). The taxonomic position of strain CMAA 1215 was further evaluated by using the JSpecies package (5).

A total of 2,717,642 assembled reads (*Q* > 20) with a mean length of 183 bp were obtained using a reference-based approach and allocated into 224 contigs ranging from 2,827 to 183,862 bp in size (50× coverage). The assembled data were analyzed by RAST (6), and the draft genome size was found to be 6,658,235 bp, comprising 6,644 open reading frames (ORFs), 64 tRNA genes, 6 rRNA genes, and a G+C content of 62.95 mol%. The genome contains 11 copies of genes usually related to plant growth promotion (6). The 16S rRNA gene analysis revealed that strain CMAA 1215 has high identity (99.9%) to the type strain of *Pseudomonas putida* (GenBank accession no. D84020). However, the average nucleotide identity (ANI) of CMAA 1215 and its phylogenetically closely related neighbor is 88.2%, which suggests that CMAA 1215 may be the nucleus of a new taxon.

### Nucleotide sequence accession numbers.

This whole-genome shotgun project has been deposited at DDBJ/EMBL/GenBank under the accession no. AVOY00000000. The version described in this paper is version AVOY01000000.

## References

[1] Myers N Mittermeier RA Mittermeier CG Da Fonseca GA Kent J 2000 Biodiversity hotspots for conservation priorities. Nature 403:853–858 10706275 10.1038/35002501

[2] Loper JE Hassan KA Mavrodi DV Davis EW II Lim CK Shaffer BT Elbourne LDH Stockwell VO Hartney SL Breakwell K Henkels MD Tetu SG Rangel LI Kidarsa TA Wilson NL van de Mortel JE Song C Blumhagen R Radune D Hostetler JB Brinkac LM Durkin AS Kluepfel DA Wechter WP Anderson AJ Kim YC Pierson LS III Pierson EA Lindow SE Kobayashi DY Raaijmakers JM Weller DM Thomashow LS Allen AE Paulsen IT 2012 Comparative genomics of plant-associated *Pseudomonas* spp.: insights into diversity and inheritance of traits involved in multitrophic interactions. PLoS Genet. 8:e1002784 22792073 10.1371/journal.pgen.1002784 PMC3390384

[3] Taketani RG Zucchi TD Melo IS Mendes R 2013 Whole-genome shotgun sequencing of *Rhodococcus erythropolis* strain P27, a highly radiation-resistant actinomycete from Antarctica. Genome Announc. 1(5):e00763-13. 10.1128/genomeA.00763-13 24072865 PMC3784785

[4] Lin SH Liao YC 2013 CISA: contig integrator for sequence assembly of bacterial genomes. PLoS One 8:e60843. 10.1371/journal.pone.0060843 23556006 PMC3610655

[5] Richter M Rosselló-Móra R 2009 Shifting the genomic gold standard for the prokaryotic species definition. Proc. Natl. Acad. Sci. U. S. A. 106:19126–19131 19855009 10.1073/pnas.0906412106 PMC2776425

[6] Aziz RK Bartels D Best AA DeJongh M Disz T Edwards RA Formsma K Gerdes S Glass EM Kubal M Meyer F Olsen GJ Olson R Osterman AL Overbeek RA McNeil LK Paarmann D Paczian T Parrello B Pusch GD Reich C Stevens R Vassieva O Vonstein V Wilke A Zagnitko O 2008 The RAST server: rapid annotations using subsystems technology. BMC Genomics 9:75. 10.1186/1471-2164-9-75 18261238 PMC2265698

